# Buffalo Medical College

**Published:** 1848-05

**Authors:** 


*Buffalo Medical College*.—The lecture session of this Institution, now in progress, will close on Wednesday, the 14th proximo, on which day degrees will be conferred upon graduates. The class in attendance numbers ninety. The council of the University have purchased a site on the corner of Main and Virginia streets, and the new edifice is shortly to be commenced. The next session commences on the last of November, and continues *five months*.

